# Gender differentials of contraceptive knowledge and use among youth – evidence from demographic and health survey data in selected African countries

**DOI:** 10.3389/fgwh.2022.880056

**Published:** 2022-11-03

**Authors:** Sibusiso Mkwananzi

**Affiliations:** Institute for Gender Studies, University of South Africa, Pretoria, South Africa

**Keywords:** contraceptive use, youth, gender, sub-Sahara Africa (SSA), knowledge - attitude - behavior, male - female distinctiveness, demographic dividend

## Abstract

Improving family planning demand and uptake has both social and economic benefits, including increasing education attainment, reducing poverty and increased participation in the labour force. Also, contraceptive use remains a key driver in Africa to facilitate demographic transition and the demographic dividend. However, numerous challenges have prevented the take-up of contraception across the continent. This is more so the case among African youth that present the lowest levels of contraceptive use in sub-Saharan Africa. Therefore, the objective of this research was to examine gender differences in contraceptive use and knowledge of sexually active young people (15–24 years) in sub-Saharan African countries. This study used data from nine countries in sub-Saharan Africa through the Demographic and Health Surveys (DHSs) of Benin, Democratic Republic of Congo, Lesotho, Namibia, Niger, Rwanda, Senegal, South Africa and Zimbabwe. Data analysis entailed frequency distributions and cross-tabulations to describe the gender-differentiated levels of contraceptive use and knowledge among youth. Additionally, logistic regression showed the gender-specific predictors of contraceptive use for African youth. Our findings present the gender-specific predictors of contraceptive use and will contribute to policy and programme formulation for African countries and organisations that promote contraceptive use.

## Introduction

Globally, 257 million women of reproductive age (15–49 years) faced unmet need or the desire to delay or prevent their pregnancy in 2021 (1). Sub-Saharan Africa (SSA) remains one of the regions in the developing world where access and utilisation of family planning services have been increasing at a slower pace than other parts of the world, despite all the previous initiatives to strengthen family planning (2–4). Specifically, sub-Saharan Africa is estimated to have higher rates of unmet need than other regions of the world, ranging between 41% and 63% (5). Therefore, investigating the reasons underlying these high levels of unmet need from this region is still required.

Nevertheless, due to numerous efforts, the average median modern contraceptive use among married women in sub-Saharan Africa increased from 3% in the period 1970 to 1984 to approximately 48% in 2021 (5, 6). However, the improvement in uptake of contraceptives has particularly been challenging among youth in sub-Saharan Africa, evidenced through rising teenage pregnancy and the highest levels of unmet need for contraception among this group (7, 8). Therefore, this study focuses on the youth demographic in desiring to overcome this challenge and creating most impact on contraceptive use levels for the SSA region.

Additionally, an interrogation of contraceptive use and knowledge among youth is important for a number of reasons. Firstly, sub-Saharan African governments acknowledge that promotion of family planning is an important step to speed up reduction of fertility rates, help protect couples' rights as well as improve maternal health (9). It has also been established that efforts aimed at improving contraceptive demand and uptake for women in SSA have both social and economic benefits, such as increasing education attainment, reducing poverty and increased participation in the labour force (2). In addition, the future of young people is particularly affected by sexual and reproductive health (SRH) outcomes, and thus, lack of access to contraception can even decrease life expectancy of girls and young women. This is because, at this stage, girls are biologically at higher risk of sexually transmitted infections (STIs), are faced with advanced childcare burdens, are more likely to experience stigma and isolation if they experience premarital childbirth, and are likely to succumb to maternal mortality in SSA, accounting for 15 percent of all mortality (10).

This is particularly concerning as almost 60% of the population in Africa is under the age of 25 (11); approximately 800 million people (12). General awareness of the social and economic benefits obtained from this youth bulge and how youth can contribute towards development (also known as the demographic dividend) exists, yet it is not clear how policy can assist in preparing the youth to meet this expectation. Thus far, the demographic dividend has not been reaped in Africa, despite the window of opportunity opening in 2000 (13). However, if family planning programmes are not effective in meeting the demand for contraception, national economies could develop a large youth dependency (14).

This study has considered analysing predictors of contraceptive use by gender. The recent COVID-19 pandemic has shown the importance of gender-sensitive social policies as crisis response requires a foundation of firm and clear social policies for effective implementation and success. While multi-level global crises affect all those living on the planet, responses and impacts are highly differentiated and exacerbate gender, class and spatial inequalities. The COVID-19 pandemic exacerbated a number of already present challenges on the continent concerned with gender. The UNFPA (2020) estimated that six months of significant health service disruptions could result in 47 million women in least developed countries (LDCs) going without contraceptives, leading to an additional 7 million unintended pregnancies (15). These concerning reproductive health outcomes underscore the need for analysing the existing evidence and data on gender-specific differentials for contraceptive use to ensure the building of relevant health systems for the youth of Africa. Addressing gender when monitoring and evaluating family planning and reproductive health projects and interventions helps to ensure equity in access and benefits for men as well as women.

Previous studies have found that knowledge of contraception methods is lowest among young women and men in SSA compared to other regions (14). Limited knowledge about sexual and reproductive health among youth is an important driver of reduced access to contraception and safe abortion services, especially among unmarried youth (16). Therefore, the first step to improving sexual and reproductive health among young people is eradicating the knowledge gap. More specifically, Hindin, Christiansen and Ferguson (2013) noted that increased knowledge around contraception was a crucial area of sexual and reproductive health among young people (17). Despite these knowledge gaps spanning gender, girls faced higher consequences, including being blamed for pregnancy or dealing with the effects of unsafe abortions (16). It has been shown that most female youth acquire information about contraception mainly from peers and family, while young men obtain contraceptive knowledge from the media, peers and pornography (14, 16). Such sources of information perpetuate myths surrounding effectiveness and side effects of various types of contraception. Therefore, this study investigated whether contraceptive knowledge among young people predicted their use to possibly advocate for further awareness.

Previous studies investigating the predictors of contraceptive use among youth in various sub-Saharan African countries found that older age, higher education levels, employment, marriage and urban residence were positive predictors for contraceptive use among females and males (8, 18–24). However, most previous studies have focused on young women or men separately and not shown the differences and similarities occurring between the sexes. This study aimed to show gender differentials for contraceptive use and knowledge to ensure that programming becomes more relevant and appropriate by gender.

**Figure 1:**
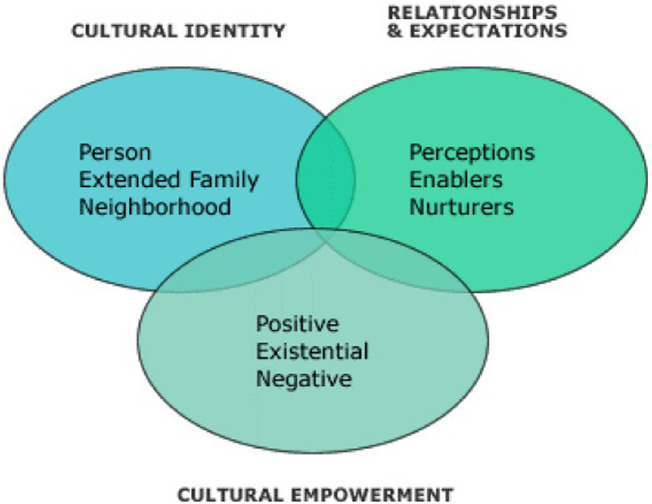
The PEN-3 cultural model from Blackstone, Nwaozuru and Iwelunmor (2018).

To interrogate the gendered predictors of contraceptive use, the study utilised the PEN-3 cultural model. Airhihenbuwa (1990) developed the PEN-3 cultural model in 1989 and updated it in 1994 (25). The model considers cultural and social factors influencing health. Its use has become popular in the design and implementation of culturally sensitive and appropriate public health interventions. As seen in Figure 1, the acronym PEN-3 illustrates the three aspects that influence health, being: (1) Cultural Identity (comprising Person, Extended Family, and Neighbourhood factors); (2) Relationships and Expectations (including Perceptions, Enablers, and Nurturers factors); and (3) Cultural Empowerment (made of Positive, Existential, and Negative factors). The model has been used in reviewing HIV, nutrition, physical activity, contraceptive use and violence in numerous developing countries, including sub-Saharan African countries (26–29).

We used the cultural identity and relationships and expectations aspects of the model to investigate person (or individual), neighbourhood, perceptions and enabler factors. This was particularly important in the African context, and countries were chosen where culture and the sense of community remain important. A full list of the independent variables included in the study is shown below in Table 1.

**Table 1:** Variable identification for the study.

Study variables	
Person/Individual Factors	
Age	15,16,17,18,19,20,21,22,23,24
Level of Education	None, Primary, Secondary, Higher
Employment Status	Unemployed, Employed
Neighbourhood Factors	
Place of residence	Urban, Rural
Relationship Factors	
Partner’s Age	Numerical value ranging from 15–91
Marital Status	Never in Union, Currently Married/Cohabiting, Formerly Married/Cohabiting
Enabler Factors	
Household size	Numerical value ranging from 1–58
Wealth Index	Poor, Middle, Rich
Knowledge of Contraceptive Methods	Knows no Method, Knows only folk/traditional method, Knows modern methods
Perception Factors	
Wife Justified to Negotiate for Safe Sex if Husband has STI	No, Yes, Don’t Know

## Data sources and methods

This study used anonymised secondary data from the most recent DHSs available of nine sub-Saharan African countries. The DHS is a cross-sectional and nationally representative survey conducted every five years that collects data on health, fertility, nutrition, mortality, as well as demographic and socio-economic characteristics for individuals and households. Countries included in this study are as follows: Democratic Republic of Congo 2014–2015, Rwanda 2019–2020, Lesotho 2014, Namibia 2013, South Africa 2016, Zimbabwe 2015, Benin 2017–2018, Niger 2012 and Senegal 2019. The selection of the countries was based on data availability as well as selecting a combination of countries with higher modern contraceptive usage (South Africa, Lesotho, Namibia, Zimbabwe), moderate levels of modern contraceptive usage (Rwanda) as well as lower modern contraceptive usage (Benin, Democratic Republic of Congo, Niger and Senegal) (18). The country data for females was obtained from responses to the woman's questionnaire, also indicated as the individual recode data, and for males was obtained from responses to the man's questionnaire, also indicated as the male recode data. These data were pooled and analysed by gender. Therefore, the study sample comprised sexually active 15–24 year olds, specifically 24,977 young women, as well as 9,377 male youth. From all respondents, individuals currently using a modern method of contraception were compared to those not using modern contraceptives.

### Variable measurement

The main dependent variable of the study was current use of modern contraceptives. To show the details of this variable clearly, univariate analysis highlighted frequencies and percentages of contraceptive use by method, where levels of non-use, folkloric/traditional methods and modern methods were shown. The study considered all 15–24 year olds who had initiated sexual activity and answered positively to using modern contraceptives, and compared these youth to their counterparts who were not using modern contraceptives at the time of the survey. Independent variables included in the study with their categories are shown in the table below:

### Statistical analysis

STATA 15 was used for data cleaning, storage and all analysis. Univariate analysis encompassed the depiction of the distribution of characteristics by gender. Levels of categorical variables were shown through frequencies and percentages, while the median and interquartile ranges of numerical variables were reported for all demographic and socio-economic measures. The gender-specific sample distribution by contraception use and knowledge variables was depicted through graphs. Bivariate analysis entailed the use of chi-squared testing for categorical factors and the non-parametric Wilcoxon test for numerical variables as they were not normally distributed. The critical value of significance was set at 0.05 for all tests conducted.

Inferential statistical analysis to determine predictors of current use of modern contraceptives involved binary logistic regression. Current use of modern contraceptives was fitted to the model, a dichotomous outcome with possible responses of “yes” and “no”. Current use of modern contraceptives was regressed on independent variables, using two models for each gender. Model one considered unadjusted demographic and socio-economic variables, followed by model two, which controlled for all variables showing adjusted odds ratios.

## Results

### Descriptive outcome

The sample characteristics of the male and female youth samples are shown in Table 2 below. The table shows that among female youth, participants had a median age of 20 years. The interquartile range was 3 years. The greatest proportion of youth females was currently married or cohabiting. More female youth lived in rural areas. For level of education, female youth predominantly had secondary education, while less than 10% of them had higher education. The majority of girls were unemployed. The median household size was 5, with an interquartile range of 4 people. Greater proportions of poor and richer girls were present, with the least numbers in the middle group. Finally, the median age for young women's partners was 28 years, with an interquartile range of 6 years.

**Table 2:** Sample characteristics by gender.

Characteristic	Females, *n* = 24,977	Males, *n* = 9377
Age (med; IQR)	20;3	20;2
Marital Status		
Never in Union	9829 (39%)	7695 (82%)
Currently Married/Cohabiting	13994 (56%)	1525 (16%)
Formerly Married/Cohabiting	1154 (5%)	157 (2%)
Place of residence		
Urban	9157 (37%)	3667 (39%)
Rural	15820 (63%)	5710 (61%)
Level of Education		
None	5506 (22%)	610 (6%)
Primary	6712 (27%)	2349 (25%)
Secondary	11917 (48%)	5878 (63%)
Higher	838 (3%)	538 (6%)
Employment Status		
Unemployed	14662 (59%)	3801 (41%)
Employed	10276 (41%)	5573 (59%)
Household size (med; IQR)	5;4	5; 5
Wealth Index		
Poor	10134 (40%)	3352 (36%)
Middle	4948 (20%)	2038 (22%)
Rich	9895 (40%)	3987 (43%)
Partner’s Age (med; IQR)	28;6	18;3

Among male youth, the median age was 20 years, with an interquartile range of 2 years. The greatest proportion of youth males was never in union and lived in rural areas. For level of education, the greatest proportion of male youth had secondary education, while the smallest proportion had higher education. The majority of young men were employed. The median household size was 5. Greater proportions of poor and richer male youth were present, with the least numbers in the middle group. Finally, the median age for young men's partners was 18 years and the interquartile range was 3 years.

Perception-related indicators revealed that the majority of female (77%) and male (84%) youth believed that it was justified for a wife to ask her husband to use a condom if he had a sexually transmitted infection (STI).

**Figure 2:**
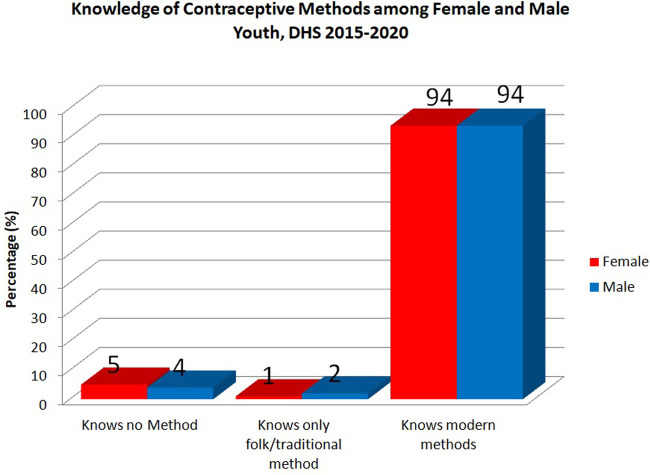
Levels of contraceptive method knowledge among youth by gender.

The levels of contraceptive method knowledge by gender are seen in Figure 2. Nearly all (94%) female and male youth knew about modern contraceptive methods. Additionally, only 5% of females and 4% of male youth knew no method of contraception, while 2% of male and 1% of female youth knew only folk or traditional methods of contraception.

**Figure 3:**
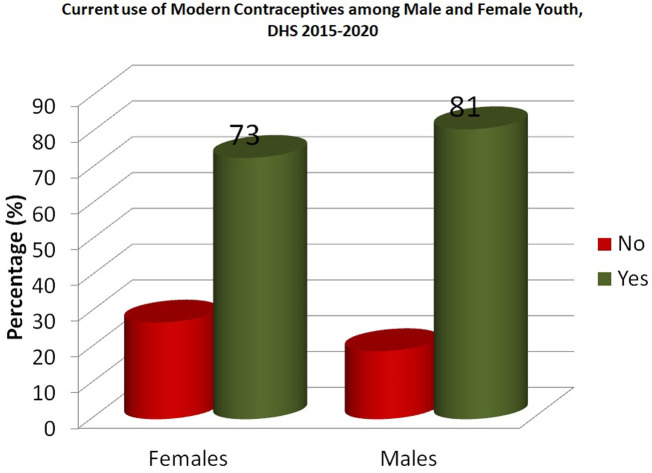
Percentage of current use of modern contraceptives by gender among youth in SSA.

Levels of modern contraceptive use can be seen for female and male youth in Figure 3. Most male and female youth reported to be current users of modern contraceptives.

Table 3 delineates the distribution of male and female youth modern contraceptive users across various characteristics. Among female youth users, the median age was 21 with an inter-quartile range of 4 years. Young women using contraceptives were predominantly never in union, living in rural areas, in possession of secondary education, unemployed and from the richer wealth index group. Their partners had a median age of 28 years old with an interquartile range of 6 years. The majority of these female youth contraceptive users agreed that wives were justified to negotiate for safe sex if their husbands had an STI and had a median household size of 5 people. The above characteristics, except partner's age, showed statistically significant differences across contraceptive use.

**Table 3:** Distribution of characteristics for users of modern contraceptives by gender.

Characteristic	Females, *n* = 7,070	*P*-value	Males, *n* = 4,622	*P*-value
Age (med; IQR)	21;4	0.000	21;4	0.000
Marital Status		0.000		0.006
Never in Union	3567 (51%)		4136 (90%)	
Currently Married/Cohabiting	3210 (45%)		424 (9%)	
Formerly Married/Cohabiting	293 (4%)		62 (1%)	
Place of residence		0.000		0.000
Urban	3168 (45%)		2131 (46%)	
Rural	3902 (55%)		2491 (54%)	
Level of Education		0.000		0.000
None	505 (7%)		108 (2%)	
Primary	1601 (23%)		1030 (22%)	
Secondary	4530 (64%)		3114 (67%)	
Higher	433 (6%)		370 (8%)	
Employment Status		0.000		0.000
Unemployed	4618 (65%)		2138 (46%)	
Employed	2441(35%)		2482 (54%)	
Household size (med; IQR)	5;5	0.000	5;5	0.000
Wealth Index		0.000		0.000
Poor	2533 (36%)		1332 (29%)	
Middle	1440 (20%)		962 (21%)	
Rich	3097 (44%)		2328 (50%)	
Partner’s Age (med; IQR)	28;6	0.611	19;3	0.000
Knowledge of Contraceptive Method		0.000		0.000
Knows no Method			24 (0.5%)	
Knows only folk/traditional method			9(0.2%)	
Knows modern methods	7070 (100%)		4589 (99.3%)	
Wife Justified to Negotiate for Safe Sex if Husband has STI		0.000		0.000
No	519 (10%)		364 (10%)	
Yes	4792 (89%)		3354 (89%)	
Don’t Know	81 (1%)		34 (1%)	

Young men using contraceptives had a median age of 21 years. Their median household size was 5 people. Male youth users had partners of a lower median age of 19 years and an interquartile range of 3 years. Young men using contraceptives were predominantly never in union, living in rural areas, with a secondary level of education, employed, from the richer wealth index category, and believed that wives were justified to negotiate for safe sex if their husbands had an STI.

### Inferential outcome

The unadjusted and adjusted regression results are shown in Table 4. Statistically significant predictors of contraceptive use among young women were age, level of education, employment status, household size and attitudes towards safe sex.

**Table 4:** Unadjusted and adjusted logistic regression of contraceptive use among youth females and males.

Characteristic	Females		Males	
	Bivariate OR (95% CI)	Multivariate OR (95% CI)	Bivariate OR (95% CI)	Multivariate OR (95% CI)
Age	1.13 (1.11–1.14)*	1.19 (1.17–1.22)*	1.06 (1.04–1.08)*	1.05 (1.02–1.08)*
Marital Status (RC-Never in Union)				
Currently Married/Cohabiting	0.52 (0.49–0.55)*	Omitted	0.33 (0.29–0.37)*	0.24 (0.20–0.28)*
Formerly Married/Cohabiting	0.60 (0.52–0.69)*	Omitted	0.56 (0.41–0.78)*	0.47 (0.32–0.69)*
Place of residence (RC-Urban)				
Rural	0.62 (0.59–0.65)*	1.01 (0.89–1.15)	0.56 (0.51–0.61)*	0.91 (0.79–1.05)
Level of Education (RC-None)				
Primary	3.10 (2.79–3.45)*	3.70 (3.18–4.31)*	3.63 (2.90–4.54)*	4.26 (3.16–5.73)*
Secondary	6.07 (5.50–6.70)*	5.45(4.69–6.33)*	5.24 (4.23–6.49)*	3.57 (2.68–4.77)*
Higher	10.59 (8.99–12.47)*	6.14 (4.17–9.05)*	10.24 (7.76–13.50)*	4.81 (3.26–7.11)*
Employment Status (RC-Unemployed)				
Employed	0.68 (0.64–0.72)*	0.64 (0.58–0.70)*	0.62 (0.57–0.68)*	0.65 (0.57–0.74)*
Household size	0.96 (0.95–0.97)*	0.98 (0.96–0.99)*	0.96 (0.95–0.97)*	0.94 (0.93–0.96)*
Wealth Index (RC-Poor)				
Middle	1.23 (1.14–1.33)*	0.92 (0.81–1.05)	1.36 (1.21–1.52)*	1.35 (1.16–1.57)*
Rich	1.37 (1.29–1.45)*	1.04 (0.91–1.18)	2.13 (1.94–2.34)*	1.87 (1.60–2.18)*
Partner’s Age	0.99 (0.99–1.00)	0.99 (0.99–1.00)	1.09 (1.07–1.11)*	1.08 (1.05–1.11)*
Knowledge of Contraceptive Method (RC-Knows no Method)				
Knows only folk/traditional method	Omitted		0.85 (0.39–1.86)	0.59 (0.25–1.43)
Knows modern methods	Omitted		17.03 (11.25–25.78)*	11.35 (7.30–17.65)*
Wife Justified to Negotiate for Safe Sex if Husband has STI (RC-No)				
Yes	2.70 (2.44–2.98)*	1.99 (1.74–2.28)*	2.04 (1.79–2.33)*	1.61 (1.36–1.91)*
Don’t Know	0.49 (0.38–0.63)*	0.57(0.41–0.80)*	0.69 (0.45–1.00)	1.10 (0.64–1.88)

*Statistically significant results that had a *p*-value of less than 0.05.

Higher age, having any form of education, and believing that wives were justified to negotiate for safe sex were positively associated with using contraceptives. Specifically, the adjusted model showed that the likelihood of contraceptive use increased by 19% as age increased by one year, almost fourfold among young women with primary education, fivefold for those with secondary education, and sixfold for tertiary educated girls compared to their counterparts with no education. Female youth that were employed had a 36% lower likelihood of contraceptive use compared to their unemployed counterparts. The odds of contraceptive use decreased by 2% as the household size increased by one person. Young women who believed that a woman was justified to negotiate for safe sex if her husband had an STI had 99% higher odds of using contraceptives, while those that were not sure if a wife was justified had a 43% lower likelihood of using contraceptives, both compared to their counterparts who believed that a wife was not justified to negotiate for safe sex if her husband had an STI.

Among male youth, the statistically significant predictors of contraceptive use were age, marital status, level of education, employment status, household size, wealth index, partner's age, knowledge of contraceptive methods and attitudes towards safe sex. Factors that were positively associated with contraceptive use included higher age, being educated regardless of level, coming from middle or richer homes, higher partner's age, knowledge of modern contraceptive methods and believing that wives were justified to negotiate for safe sex. In particular, the likelihood of contraceptive use increased by 5% for every one-year increase in age. Married or cohabiting young men had a 76% lower likelihood, and formerly married or cohabiting young men a 53% lower likelihood of contraceptive use compared to their never-in-union counterparts. The odds of contraceptive use increased fourfold among young men with primary education, almost fourfold for those with secondary education and almost fivefold for tertiary educated boys compared to their counterparts with no education. Male youth that were employed had a 35% lower likelihood of contraceptive use compared to their unemployed counterparts. The odds of contraceptive use decreased by 6% as the household size increased by one person. Young men living in the middle households had 35% higher odds of contraceptive use, while those living in the richer households had 87% higher odds of contraceptive use compared to young men living in the poorer households. As the partner's age of young men increased by a year, their likelihood of contraceptive use increased by 8%, knowledge of modern contraceptive methods increased the odds of contraceptive use 11 times and, finally, male youth who believed that a woman was justified to negotiate for safe sex if her husband had an STI had 61% higher odds of using contraceptives compared to their counterparts who believed that a wife was not justified to negotiate for safe sex if her husband had an STI.

## Discussion

This study aimed to show the levels, differentials and predictors of contraceptive use among male and female youth in sub-Saharan Africa. Significant differences were shown across gender in contraceptive use, with female youth having lower levels at 28%, while it was positively higher at 49% among male youth. Previous studies in the African setting have shown similar prevalence of contraceptive use. Ahinkorah (2020) showed that approximately one in four young women used contraceptives, and Mandiwa et al. (2018) found that 31% of young women used modern contraceptives (18, 20). Additionally, Oyediran (2011) established that 20% of young men were using modern contraceptives at sexual debut (24). The current study's distinct difference in male and female youth levels of contraceptive use indicates that these two demographic counterparts do not necessarily correlate with each other. This can be seen in the difference in median for partner's age. Among young women, the median partner's age was 28 years old, while partner's age was 18 years old among young men. Female youth's partners being older and male youth having younger partners follows the general convention in the African context of women being in relationships with men that are their peers or older.

The study found that increasing age was positively associated with the use of contraceptives. This was seen among female and male youth. Our results for young women concur with previous studies from numerous countries in sub-Saharan Africa. Specifically, studies from Ethiopia, Malawi and Ghana as well as a systematic review, including multiple sub-Saharan African countries, all showed that the likelihood of contraceptive use increased with age among young women (18–20, 22, 23). However, previous studies investigating predictors of contraceptive use among men did not find an association with age (8, 21, 24).

Possible explanations for this association include that, as age increases, experience and maturity increase as well. This means that young women may find it easier to negotiate for safe sex as they get older, whereas, when younger, they may be intimated by their male partners as well as the need to talk about sexuality and safe sex. Seutlwadi et al. (2012) demonstrated that talking to a partner about condom use was associated with contraceptive use (8). However, Kriel, Milford, Cordero, Suleman, Beksinska, Steyn and Smit (2019) revealed the power dynamics involved in relationships that affect contraceptive use, and concluded that an important reason of male opposition to contraceptive use was male dominance in the relationship (30). This male dominance could be less challenging when the female partner is older. The responsibility of one's own sexuality and the consequences involved in not using contraceptives may be realised and taken more seriously as one becomes older. This explanation was further emphasised by the positive association found among men between their partner's age and the use of contraceptives, showing that negotiation for safe sex and the insistence on contraceptive use increased as a man's partner increased in age.

Additionally, higher age is linked to higher levels of education, largely among young people in Africa. This is despite concerns of high dropout and grade repetition in numerous African countries. Although age and education were not correlated in this study, previous studies have shown a strong correlation between age and level of education. This was further confirmed by the cross-tabulation of age categories and education levels that showed that the majority of 20–24-year-old female and male youth had secondary and higher education, while 15–19 year olds predominantly had primary and secondary education. In addition to this link through age, the current study, like previous research, showed that higher levels of education were independently associated with an increased likelihood of contraceptive use for male and female youth. Studies from Malawi and Ghana for young women, as well as Kenya and South Africa for men, aligned with our findings in this regard, but Ngome and Odimegwu (2014) in Zimbabwe found no association between education and contraceptive use among female youth (8, 20, 22–24). Higher levels of education would mean that individuals would have higher health literacy and exposure to information, and could read as well as find out about contraceptives for themselves. In addition, they could explore health and contraception information from books, magazines and the internet, all of which could then assist them in making personal sexual and reproductive health and rights (SRHR) decisions by themselves. Therefore, keeping young people in school for as long as possible is once again shown in this study as an important element in ensuring higher contraceptive use and thus lower levels of fertility.

Youth that were employed had a lower likelihood of contraceptive use among males and females. Employment has also been shown to be negatively related to contraceptive use in a review of contraceptive use among young women (18). However, most previous studies have found a positive association between employment and contraceptive use for male and female youth (8, 20, 23). A possible reason for the negative association found would be that working individuals are less worried about having a child because they have the means to take care and provide for that child. However, the risk of a child not being cared for adequately is exacerbated when individuals are unemployed, resulting in a higher use of contraceptives in this group. This would be facilitated by unemployed individuals having the time to obtain contraceptives at the health facility. Conversely, employed individuals may not have the time to attend health facilities to obtain contraceptives because of the need to be at work.

The research depicted that marital status was only a predictor of contraceptive use among male youth. Study results from Kenya and Ethiopia were similar to this study (31, 32). Explanations for this finding may be that married men and formerly married men are not concerned about having children. This is because having children is normalised in a setting of marriage and in cohabitation. Only among single/never-in-union males does there seem to be a perceived risk of impregnating someone and thus the need to use contraceptives. Additionally, marriageability among men is higher among those that are working and, as seen from our previous finding regarding employment, this group tended to have lower odds of using contraceptives. A cross-tabulation of marital status and employment among men showed that the highest percentage of employed men were married. The association depicted here is indicative of married and cohabiting men making SRHR decisions for their partners in these relationships as well as the familial and societal pressure placed on such couples to have children.

This finding is important in showing the need for contraception promotion programmes to target married and cohabiting men rather than women. Women are constantly inundated with education materials, awareness campaigns and other programmes to increase their uptake of contraceptives, and yet this finding and the fact that marital status was not significantly associated with contraceptive use among women gives clues as to why this approach has been largely unsuccessful. These results are related to that of knowledge of modern contraceptives increasing the use of contraceptives elevenfold among young men. Previous South African research aligned with this finding when it established that greater contraceptive knowledge was associated with contraceptive use among men in univariate logistic regression (8). This speaks to the importance of awareness, campaigns and knowledge transfer that promotes modern contraceptive use aimed at men specifically. The result being significant among men and not women again points to the fact that the emphasis of family planning awareness drives should be on men and not women, who largely know about modern contraceptives without linked behaviour in use. Therefore, it is imperative to establish how males as partners promote and assist in adherence and use of contraceptives, and how these insights can be integrated into policy to improve the uptake and use of family planning (30). Therefore, as advocated by previous literature, men should be involved in the SRHR of women, and a focus placed on men rather than women through programming (33).

The research established that household size was negatively associated with contraceptive use for young men and women. Household size refers to the number of people in the home. This relationship may be explained firstly through household size being linked to wealth status. Larger households may be poorer, and among the poor, the levels of contraceptive use are also lower. This was confirmed through the cross-tabulation of household size categories and wealth status, which showed that the majority of the largest households had poor and medium wealth status, while smaller households predominantly displayed medium and richer wealth status. In such households, having a child has fewer consequences for an individual's future prospects because opportunities are very limited as well. Therefore, pregnancy and being a father in such settings would not jeopardise any hope of attending tertiary education or being well employed, as opposed to their counterparts in rich households.

Larger households may also be more traditional, with beliefs and attitudes against the use of contraceptives and termination of pregnancy. Therefore, it may be normalised in such households that “children are a blessing” and “children are from God”, making the decision to prevent or terminate a pregnancy more unlikely. The perception of the risk of pregnancy in such settings is thus lower. The importance of perceptions is seen in the result that attitudes towards justification for safe sex were positively associated with the use of contraceptives among young men and women. This is indicative of perceptions being predictors of behaviour. Therefore, the perceptions of individuals usually influence their actions. This finding suggests that larger households should be targeted for awareness campaigns and increased education drives on contraceptive methods and termination of pregnancy to encourage the use of contraceptives. Such programmes may be beneficial for these households if they were linked to grants or social transfers that encouraged contraceptive use. Such financial incentives could be used to start small businesses, pursue further education or acquire skills.

Among young men, as wealth status increased, the likelihood of use also increased. These results aligned with previous research from Nigeria, Kenya and Uganda (24, 31, 34). This may be explained once again with the risk of impregnating someone being higher when your future prospects are better, as among men from richer households. Individuals from higher socio-economic status households usually have better access to and knowledge of contraceptives as well as a higher ability to negotiate and enforce safe sex with their partners. Studies have shown that youth from poor homes are more likely to have premarital births than those from affluent backgrounds (35–39).

Wilson (1991) clarified that poor areas lack educated, employed and married role models; and the lack perpetuates social welfare reliance and family instability as norms (40). This leads to poor families giving up hope of ever overcoming economic hardship for themselves or their children. Schooling and employability may be so weak in such areas that staying in school and avoiding pregnancy through using contraceptives may not be advantageous (41). Accordingly, families living in poverty may adopt practices that are less conducive to school and career success while discouraging contraceptive use (42).

Findings from this study can be used for family planning programming specifically aimed at youth across sub-Saharan Africa. Now more than ever, it is important to prioritise SRHR in health systems to prevent high levels of fertility, promote bodily autonomy, and ensure people have access to lifesaving health services**.** Strengthening of SRHR services and an increase in uptake of contraceptives among young people would also assist in attaining the demographic dividend in sub-Saharan Africa. Most sub-Saharan African countries have focused on employment for young people without ensuring preliminary and complementary investments in governance, family planning, maternal and child health, education, and women's empowerment, which would secure a productive labour market (43). Although dependency ratios and fertility in sub-Saharan Africa remain high, they have started to decrease. According to UN projections, they will fall further in the coming decades, such that by the mid-21st century the ratio of the working-age to dependent population will be greater than in Asia, Europe, and Northern America (44).

Research shows that social norms that oppose contraceptive use as well as those that are gender biased tend to dissuade female labour force participation, leading to low foregone benefits in having children and thus continued high fertility. High fertility also leads to low investment in the education, upskilling and human capital of children (44). Regarding actionable steps towards fertility reduction, Bloom et al. (2013) show that fulfilling just one third of the unmet need for contraceptives in Kenya, Senegal, and Nigeria could lead to an increase in per capita income of 8%–13%, while an increase of 31%–65% in per capita income could be seen by meeting all of the unmet need in contraceptives (45). Therefore, creating a favourable policy environment for generating and capitalising on a demographic dividend, can support sub-Saharan Africa's stated goal for development.

This observation points to the value of policies aimed at overcoming the social norms underlying persistently high fertility, including educational campaigns, facilitating female labour market access (even against a norm), reducing labour market discrimination against women as well as creation or expansion of marketplaces to dampen fertility by enhancing information exchange on modern methods of contraceptives (44). It is hoped that the findings from this study will work towards the implementation of contraception programmes aimed at the youth being more relevant, accessible and successful.

## Data Availability

Publicly available datasets were analyzed in this study. This data can be found here: https://dhsprogram.com/Data/.
